# A case of dystonia with polycythemia and hypermanganesemia caused by SLC30A10 mutation: a treatable inborn error of manganese metabolism

**DOI:** 10.1186/s12887-019-1611-7

**Published:** 2019-07-09

**Authors:** Azita Tavasoli, Khadije Arjmandi Rafsanjani, Saba Hemmati, Marziyeh Mojbafan, Elham Zarei, Soudabeh Hosseini

**Affiliations:** 1 Department of Pediatric Neurology, Ali Asghar Children’s Hospital, Iran University of Medical Sciences, Tehran, Iran; 2 Department of Pediatric Hematology, Ali Asghar Children’s Hospital, Iran University of Medical Sciences, Tehran, Iran; 3 Department of Pediatrics, Ali Asghar Children’s Hospital, Iran University of Medical Sciences, Tehran, Iran; 4 Department of Medical Genetics and Molecular Biology, Faculty of Medicine, Iran University of Medical Sciences, Tehran, Iran; 5 Department of Radiology, Ali Asghar Children’s Hospital, Iran University of Medical Sciences, Tehran, Iran; 6 Ali Asghar Children’s Hospital, Iran University of Medical Sciences, Tehran, Iran

**Keywords:** Case report, Dystonia, Hypermanganesemia, Polycythemia, SLC30A10

## Abstract

**Background:**

Manganese is a critical trace element that not only has antioxidant properties, but also is essential for various metabolic pathways and neurotransmitters production. However, it can be toxic at high levels, particularly in the central nervous system. Manganese intoxication can be acquired, but an inherited form due to autosomal-recessive mutations in the SLC30A10 gene encoding a Mn transporter protein has also been reported recently. These mutations are associated with significant failure of manganese excretion and its storage in the liver, brain (especially basal ganglia), and other peripheral tissues, resulting in toxicity.

**Case presentation:**

A 10-year-old boy from consanguineous parents presented with a history of progressive truncal instability, gait difficulty, and frequent falls for 2 months. He had dystonia, rigidity, ataxia, dysarthria, bradykinesia and a plethoric skin. Investigations showed polycythemia, low serum iron and ferritin levels, and increased total iron binding capacity. A brain MRI revealed symmetric hyperintensities in the basal ganglia and dentate nucleuses on TI images that were suggestive of brain metal deposition together with clinical manifestations. Serum calcium and copper levels were normal, while the manganese level was significantly higher than normal values. There was no history of environmental overexposure to manganese. Genetic testing showed a homozygous missense mutation in SLC30A10 (c.C1006T, p.His336Tyr) and Sanger sequencing confirmed a homozygous state in the proband and a heterozygous state in the parents. Regular treatment with monthly infusions of disodium calcium edetate and oral iron compounds resulted in decreased serum manganese and hemoglobin levels to normal values, significant resolution of MRI lesions, and partial improvement of neurological symptoms during 6 months of follow-up.

**Conclusion:**

The syndrome of hepatic cirrhosis, dystonia, polycythemia, and hypermanganesemia caused by SLC30A10 mutation is a treatable inherited metal deposition syndrome. The patient may only have pure neurological without hepatic manifestations. Although this is a rare and potentially fatal inborn error of metabolism, early diagnosis and continuous chelation therapy might improve the symptoms and prevent disease progression.

## Background

Manganese (Mn) is a critical trace element that not only has an antioxidant activity, but also is essential for various metabolic pathways [1]. It is necessary as a cofactor for several enzymes in the metabolism of neurotransmitters and hormones [2]. Moreover, Mn competes with other ions for protein binding and its excess amounts can lead to oxidative stress, mitochondrial dysfunction, DNA replication disturbance, and apoptosis, particularly in the brain [3]. Moreover, metabolic pathways of several neurotransmitters including dopaminergic ones could be directly damaged by Mn accumulation in the basal ganglia. The Mn level in the body is tightly regulated through the physiologic control of its intestinal absorption and biliary excretion via a Mn transporter protein encoded by SLC30A10 [4]. Mn intoxication can be seen in acquired situations such as environmental overexposure, chronic liver disease leading to excretion failure, drinking contaminated water, receiving prolonged parenteral nutrition, and in some cases of drug abuse [5]. Excess Mn storage in the liver and brain, especially in the basal ganglia, results in hepatic cirrhosis and neurological disturbances characterized by behavioral problems and an extrapyramidal syndrome [6].

Autosomal-recessive mutations in the SLC30A10 gene can be associated with a significant failure of Mn excretion leading to its storage in the liver, brain, and other peripheral tissues [7]. Here we report a 10-year-old boy who presented with dystonia, polycythemia, hypermanganesemia and typical brain findings on the MRI. Genetic testing showed a homozygous SLC30A10 mutation. To the best of our knowledge, this is the first Iranian report of this rare, treatable, hereditary metal storage disorder.

### Case presentation

The patient was a previously healthy 10-year-old boy presenting with progressive truncal instability, gait difficulty, and frequent falls from 2 months ago. He was born to healthy consanguineous Balooch parents, an ethnic group living in the southeast of Iran. His birth history was uneventful but a mild motor developmental delay was reported as he started to walk independently at the age of 2 years. Family history was negative for neurological or hematological diseases. The cognitive function was intact and his academic performance was average as he went to school until one month ago. On neurological examination, he had dysarthria, slowing of vertical saccades, ataxia, generalized rigidity (more dominant in lower limbs) and bradykinesia. Moreover, he had sustained four-limb dystonia, predominantly in the lower limbs, that was exacerbated by voluntary movement and fluctuated in severity over days. These twisting movements together with increased stiffness led to an abnormal posture. Lower limb dystonia increased during walking resulting in walking difficulty and a specific “cock-walk” gait. On systemic examination, his face and palms were plethoric but no other remarkable findings were noted.

**Fig. 1:**
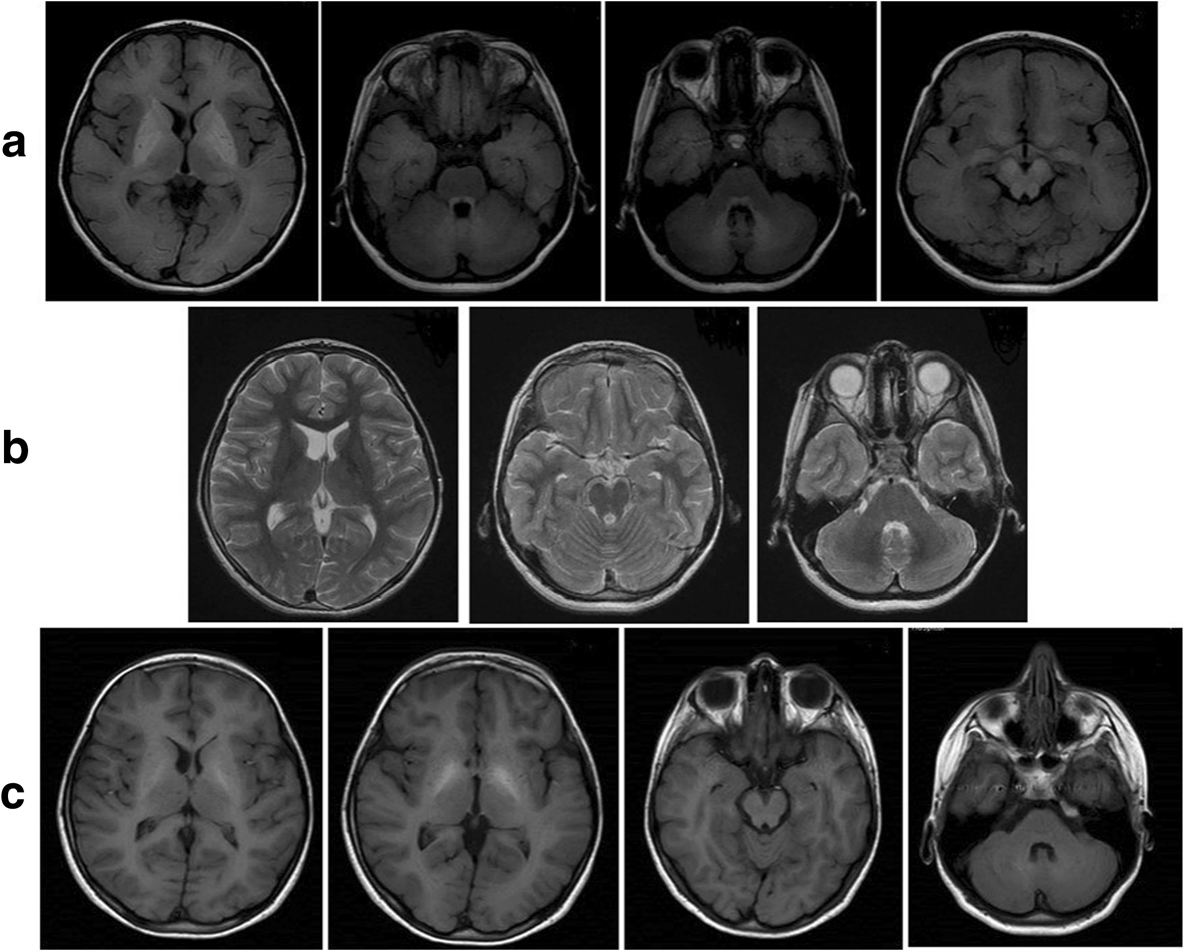
Brain MRI of the patient showed symmetric hyperintensities of the basal ganglia, tectum of the pons, and dentate nucleuses on T1- W images (**a**) and a normal appearance on T2-W images (**b**). Repeated brain MRI at 6 months follow-up showed significant resolution of previous findings on T1 images (**c**)

Laboratory investigations showed polycythemia (red cell count: 10.73 × 10^3^ / L, normal range [n.r.]: 4.1–5.3 × 10^3^; hemoglobin: 20.1 g/dl, n.r.: 12–16; hematocrit: 67.1%, n.r.: 37–47), low serum ferritin (7 ng/ml, n.r.: 12–300), and a high total iron binding capacity (TIBC: 630 mcg/dl, n.r.: 250–450). Serum calcium, liver transaminases, ammonia, lactate, and pyruvate levels were within normal limits and amino acid chromatography was unremarkable. A diagnosis of Wilson disease was suspected according to the extrapyramidal symptoms; however, serum copper and ceruloplasmin and 24-h urine copper levels were normal, and abdominal ultrasound was unrevealing. Ophthalmologic examination was unremarkable for the Kayser- Fleischer ring. Echocardiography, electromyography, and nerve conduction velocities of the limbs were normal. A brain MRI showed symmetric hyperintensities in the caudate and lentiform nucleuses, tectum of the pons, and dentate nucleuses of the cerebellum on T1 images and a normal appearance on T2 images without restriction on diffusion weighted images (Fig. 1 a,b). Neuroimaging findings were suggestive of metal deposition in the brain. As serum copper and calcium levels were normal, the serum manganese level was checked in two different laboratories by gas-chromatography and mass spectrometry (GC/MS) Agilent technology (Calif, USA). The serum level of manganese was above 3000 nmol/L, which was far beyond the reference value (less than 320). There was no history of environmental overexposure; therefore, an inherited hypermanganesemia was suggested. Key genes implicated in Mn homeostasis in humans are SL39A14, SL30A10, and SLC39A8 with 16, 6, and 16 exons, respectively [2]. Sequencing all of these exons by Sanger sequencing is difficult, expensive, and time-consuming. Therefore, whole exome sequencing was done, which showed a homozygous missense variant in SLC30A10 (c.C1006T, p.His336Tyr). According to the Sherloc comprehensive variant classification, this variant may be classified as pathogenic because of at least 6 pathogenic points (PM_2_:1 point; PP_3_: 0.5; PS_3_:2.5; PP_4_:2). Sanger sequencing confirmed a homozygous and heterozygous state in the proband and parents, respectively. The pathogenic variant was not revealed in his younger asymptomatic brother.

**Fig. 2:**
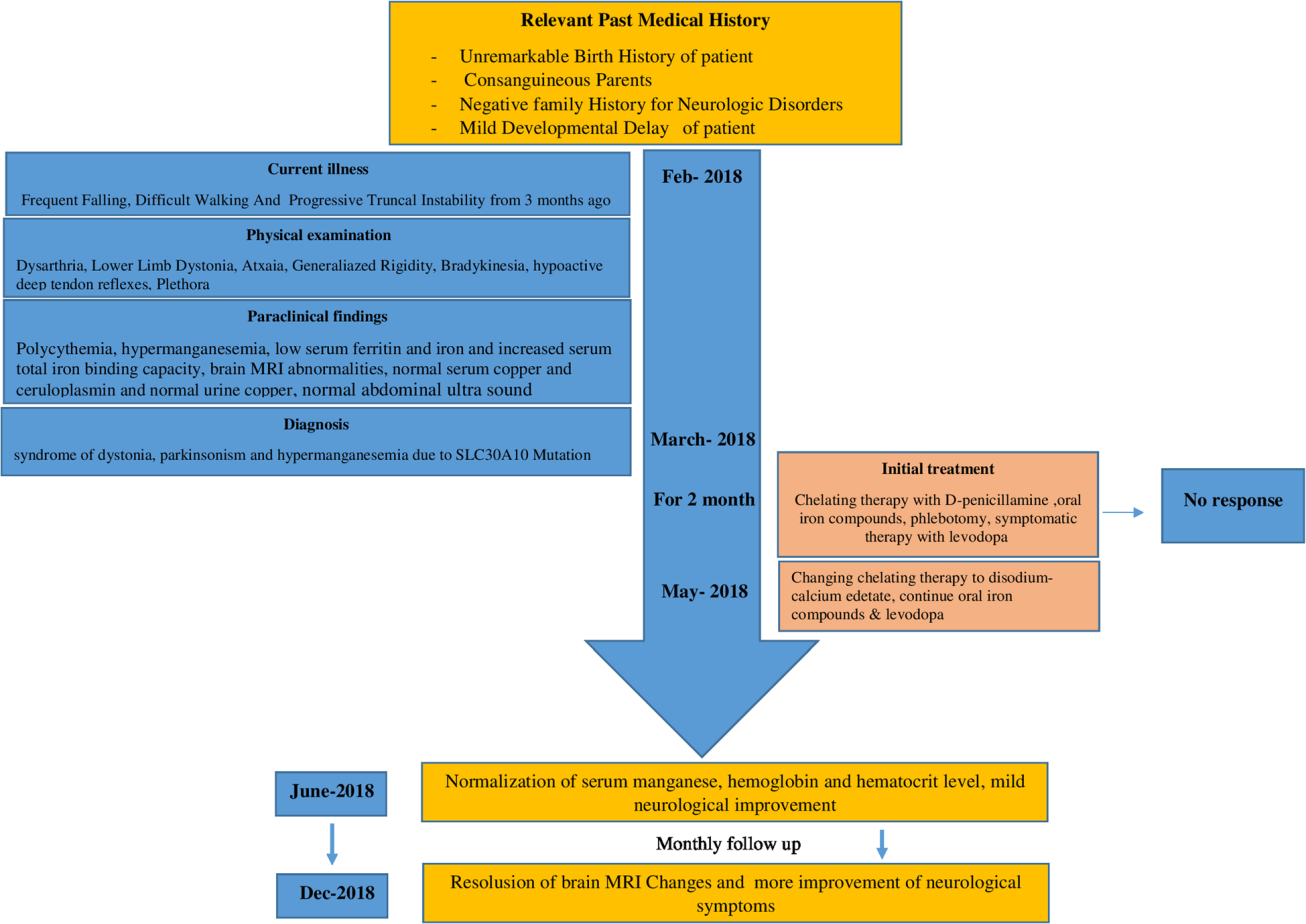
Clinical course of the patient

Symptomatic therapy with Levodopa at an optimal dosage (10 mg/kg/day in three divided doses) was not effective. A combination of oral D-penicillamine 1000 mg daily (250 mg every 6 h), iron supplementation and levodopa (300 mg daily) was started and phlebotomy was done, but no clinical or laboratory responses were noted after 2 months. Then, disodium calcium edetate was administered as a chelation therapy at 1 g/m2 for 5 days infused every 4 weeks and oral iron compounds and levodopa were continued. After two cycles of treatment, the Mn level and Hb concentration decreased to 170 nmol/L and 14 g/dl respectively, and the serum level of ferritin increased, but minimal improvement was seen in motor function. At the 6th month of follow up, complete resolution of dysarthria and partial amelioration of bradykinesia, rigidity, and dystonia were seen resulting in a better motor function and gait. Repeated brain MRI revealed marked resolution of previous findings (Fig. 1c). The Mn and hemoglobin levels were in the normal range and the motor function improved steadily throughout the follow-up period until the time of preparing this report. No adverse effects of disodium calcium edetate such as hypocalcemia, thrombocytopenia, leukopenia and renal or hepatic dysfunction were seen. The clinical course of the patient is shown in Fig. 2.

## Discussion and conclusions

A genetic form of severe hypermanganesemia secondary to SLC30A10 mutation has been recently identified [8]. We reported the clinical course, brain MRI findings, and treatment response of a patient with dystonia, polycythemia, and hypermanganesemia due to this homozygous mutation. This variant is not a novel one and was previously reported by Tuschl et al. [5] with a CADD score of 28.8. It is not reported in gnomAD (neither homozygous nor heterozygote state) but has been reported once in a heterozygous state in the ExAC database. The protein encoded by SLC30A10 is a significant Mn efflux transporter localized in the cell membrane that decreases cellular Mn levels and protects tissues against its toxicity [9]. It is highly expressed in the liver, as an important organ in the manganese homeostasis. Liver disease develops with a variable intensity and presents as hepatomegaly, increased serum transaminases, direct hyperbilirubinemia, and cirrhosis. However, it is not pathognomonic of the SLC30A10 mutation syndrome and pure neurological symptoms without hepatic manifestations have been reported [10]. A recent study showed that severity and age of onset of either neurological or hepatic dysfunction varied even among the same family members [7]. Mukhitar et al. reported liver dysfunction only in one of the three siblings with SLC30A10 mutation [3]. Quadri et al. also reported three affected siblings of whom two with severe dystonia did not have hepatic dysfunction while their sister with minor neurological symptoms died due to hepatic involvement [10]. We did not find any evidence of hepatic involvement in the proband from the disease onset through the following period. The SLC30A10 carrier protein is also highly expressed in the brain, especially in the basal ganglia, where it prevents the neurotoxic effects of manganese excess. The toxic effects of hypermanganesemia are severe and result in cumulative and chronic brain damage [4]. According to several studies, the most common neurological manifestation of hypermanganesemia is dystonic movement disorder. Tuschl et al. found that most affected individuals, particularly those affected in childhood, had walking difficulties and fine motor impairment due to dystonia [5]. We found rigidity and dystonia in our patient that resulted in walking difficulties and an unstable high stepping gait, Fine motor dysfunction led to a bad handwriting and dysdiadochokinesis, and tongue dystonia resulted in dysarthria. Gospe et al. reported spastic paraparesis in one patient [11]. Although cognitive defects and psychological symptoms have been shown in patients with acquired hypermanganesemia [12], they have not yet been reported in SLC30A10 mutations. Several studies have found that these patients have typical brain MRI findings including symmetric hyperintensities of the basal ganglia and dentate nucleuses on T1- weighted and normal appearance or milder changes as related hypointensities on T2-weighted images [3–5]. This is in contrast to Wilson disease in which a brain MRI often shows basal ganglia hyperintensities on T2-images due to copper deposition [7]. Brain MRI findings are identical in both acquired and inherited forms of hypermanganesemia [3].Other inherited syndromes with metal deposition observed on neuroimaging include brain iron accumulation syndromes that cause specific patterns of iron deposition on T2 images, and syndromes of brain calcium storage that present as hyperdensities on a CT scan [13]. Several laboratory tests help to diagnose inherited hypermanganesemia and to differentiate it from the acquired type and other inherited metal deposition syndromes. The mean serum Mn level in these patients is above 2000 nmol/L while it is lower than 2000 in acquired hypermanganesemia [13]. Moreover, polycythemia is a general finding in SLC30A10 mutation which serves as an early disease marker [7]. It may exist prior to neurological symptoms and hence recurrent phlebotomies are often recommended before making a correct diagnosis [5, 10]. Mn is known to upregulate erythropoietin gene expression, which could be a probable mechanism [4]. The mean hemoglobin concentration has been reported at 19 g/dl. A few studies have shown increased erythropoietin levels in some affected individuals [5, 10, 11]. Polycythemia can be corrected by chelation therapy or iron compounds [5]. Further findings include low serum ferritin and iron levels and increased TIBC. Iron store depletion is due to competitive inhibition of intestinal iron absorption exerted by Mn. The homeostatic mechanism of iron and Mn is closely related due to the same serum binding and transporter proteins [7]. Iron discharge from intracellular reserves, increased iron uptake, and reduced iron utilization by hypermanganesemia have been proposed as other mechanisms [14]. Reduced iron stores or polycythemia are not usual findings in acquired hypermanganesemia [4]. SLC30A10 variations may be the cause of susceptibility of some individuals to Mn toxicity in overexposure situations [10].

Lifelong chelation therapy combined with iron supplementation is the treatment of choice that should be started as soon as possible [3, 5, 15]. Intravenous disodium calcium edetate increases urinary excretion of Mn. Iron compounds prevent further intestinal absorption of Mn and can also improve polycythemia. Continuous treatment with edetate can decrease the serum Mn level and resolve brain MRI changes, bringing liver damage to a stop and causing a relative neurological improvement [4]. However, patients with acquired hypermanganesemia may show variable responses to this treatment [13]. The patients left untreated may die from cirrhosis or become severely disabled and wheelchair- bound [13].Similar to other reports, our patient showed a poor therapeutic response to levodopa. There are different reports of the effects of other chelator agents such as D-penicillamine [3, 4]. Mukhtiar et al. found beneficial effects in one patient with milder symptoms, while Stamelous et al. did not report any responses to D-penicillamine, which was similar to our case [3]. There is a need for further investigations. Since the disease manifestations are preventable, it is rational for the siblings of a patient to be assessed either by genetic testing or by regular measurement of serum Mn and hemoglobin levels.

Clinical manifestations of SLC30A10 mutation syndrome range from childhood-onset to adult-onset forms. Neurological manifestations are typically extrapyramidal in the early-onset form starting between 2 and 15 years of age. Our patient falls into the spectrum of SLC30A10 mutation syndrome with regard to the age at onset, clinical phenotype, and disease course. A firm genotype-phenotype correlation cannot be observed in this syndrome due to the small number of known patients worldwide. However, a pathogenic variant causes a premature stop codon and produces a protein lacking the last 49 amino acids leading to the adult- onset type of the syndrome [10]. Reporting other patients helps to identify the complete phenotypical spectrum of the SLC30A10 mutation syndrome.

In conclusion, we would like to highlight a syndrome caused by SLC30A10 mutation, which, along with the Wilson disease, is the only treatable inherited metal deposition syndrome. It is noticeable that the patient may only exhibit pure neurological symptoms without hepatic manifestations. Early diagnosis and genetic testing are necessary in children with early onset dystonia and typical MRI findings, especially in association with polycythemia and hypermanganesemia. Early treatment might improve the symptoms and prevent the progression of this potentially fatal disease.

## Data Availability

The data of the patients are available from the corresponding author on reasonable request.

## Abbreviations

CADD: Combined annotation dependent depletion
ExAC: Exon aggregation consortium
Gnom AD: Genome aggregation database
Mn: Manganese
MRI: Magnetic resonance imaging
TIBC: Total iron binding capacity
